# Accuracy of 3D printed spine models for pre-surgical planning of complex adolescent idiopathic scoliosis (AIS) in spinal surgeries: a case series

**DOI:** 10.1016/j.stlm.2023.100117

**Published:** 2023-08

**Authors:** Abir Dutta, Menaka Singh, Kathryn Kumar, Aida Ribera Navarro, Rodney Santiago, Ruchi Pathak Kaul, Sanganagouda Patil, Deepak M Kalaskar

**Affiliations:** a UCL Institute of Orthopaedic & Musculoskeletal Science, Division of Surgery & Interventional Science, University College London, Royal National Orthopaedic Hospital, Stanmore, HA7 4LP, London, United Kingdom; b Royal National Orthopaedic Hospital NHS Trust, Spinal Surgery Unit, Stanmore, HA7 4LP, London, United Kingdom; c Department of Radiology, Royal National Orthopaedic Hospital, Stanmore, United Kingdom

**Keywords:** Adolescent idiopathic scoliosis, 3D Printing, 3D Reconstruction, Surgical planning, Additive manufacturing

## Abstract

Adolescent idiopathic scoliosis (AIS) is a noticeable spinal deformity in both adult and adolescent population. In majority of the cases, the gold standard of treatment is surgical intervention. Technological advancements in medical imaging and 3D printing have revolutionised the surgical planning and intraoperative decision making for surgeons in spinal surgery. However, its applicability for planning complex spinal surgeries is poorly documented with human subjects. The objective of this study is to evaluate the accuracy of 3D printed models for complex spinal deformities based on Cobb angles between 40° to 95°.This is a retrospective cohort study where, five CT scans of the patients with AIS were segmented and 3D printed for evaluating the accuracy. Consideration was given to the Inter-patient and acquisition apparatus variability of the CT-scan dataset to understand the effect on trueness and accuracy of the developed CAD models. The developed anatomical models were re-scanned for analysing quantitative surface deviation to assess the accuracy of 3D printed spinal models. Results show that the average of the root mean square error (RMSE) between the 3DP models and virtual models developed using CT scan of mean surface deviations for the five 3d printed models was found to be 0.5±0.07 mm. Based on the RMSE, it can be concluded that 3D printing based workflow is accurate enough to be used for presurgical planning for complex adolescent spinal deformities. Image acquisition and post processing parameters, type of 3D printing technology plays key role in acquiring required accuracy for surgical applications.

## Highlights

• Surgical planning for Adolescent idiopathic scoliosis (AIS) is very complex.
• 3D printed model provides an excellent opportunity for surgical planning.
• Accuracy of 3D printed model for complex for AIS planning is not reported before.
• Case series, evaluates accuracy of 3D printed models for varying spinal deformities indicated by Cobb's angle (as the 40° to 95°)
• Accuracy of surgical model is dependent on Imaging, post processing parameters & 3D printing technology.

## Graphical abstract

**Figure:**
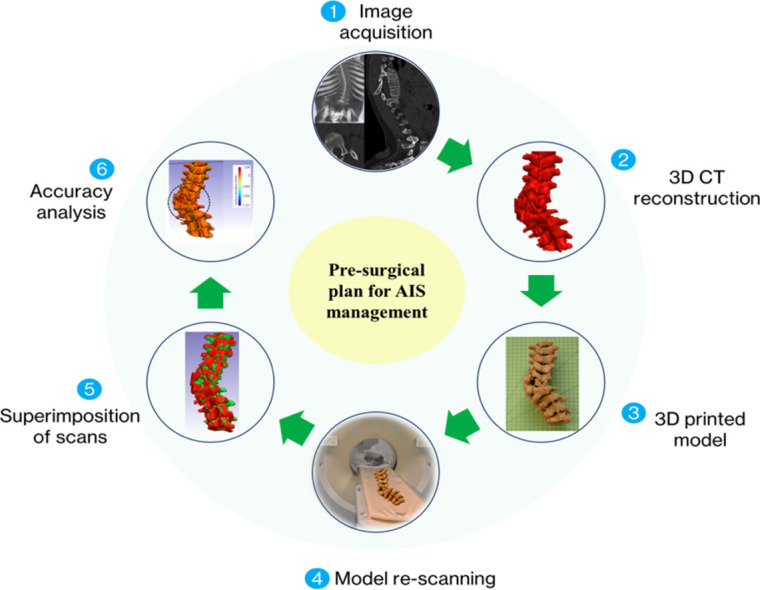

## 1 Introduction

Scoliosis is a three-dimensional (3D) structural deformity of spine with symptoms including pain, psychological morbidity and cardiorespiratory dysfunction [1]. The most common indication for surgical intervention in spinal deformity is adolescent idiopathic scoliosis (AIS) [2]. Although 80% of all scoliosis cases are classified as idiopathic, the current view is that AIS is likely a multifactorial disorder with genetic predisposition [3]. The prevalence of AIS is estimated to be between 1-3% affecting children between 10 -16 years of age (adolescent). AIS is defined by a spinal curvature of 10 degrees in the coronal plane usually affecting females [4,5]. Enlarged Cobb angles present a lower frequency with curves greater than 40 degrees constituting 0.1% of the AIS population [2]. The principles of scoliosis management have remained unchanged. A Cobb angle of 45-50 degrees indicates the need for surgery, hence a high risk of progressive deterioration into adulthood [6,7].

Surgical intervention aims to reduce pain associated with progression of the deformity and thereby stabilising the curve to reduce the impact on internal organs [8,9]. Pedicle screws are commonly used to achieve a three-column fixation of the spine, despite the wide variation in the pedicle anatomy [10]. The risks and complications associated with pedicle screws (poly axial or mono axial) include perforation of the bony cortex from incorrect screw fixation with the reported incidence of misplaced screws ranging from 3% to as high as 25% [11], [12], [13].

Accurate placement of pedicle screws is crucial to avoid complications in spinal procedures. Thus, the surgeon may have to use intra-operative fluoroscopy, which is an imaging technique using the X-rays to obtain real-time images of the reconstructive metallic device several times during a surgery to aid precise pedicle screw placement. This incurs an increased radiation exposure to the patient and increases the intra-operative time [14,15]. Surgical complications in spinal procedures can be potentially devastating due to the proximity of vital structures. Most common complications include neurological injury or wound infection [13], [14], [15], [16]. Vigorous planning in the pre-operative stages can aid the surgical team to anticipate and minimise risks in such cases.

The advancement in the quality and spatial resolution of medical imaging has made it possible to create virtual 3D reconstruction of complex anatomy from computed tomography (CT) and magnetic resonance imaging (MRI), using various image processing techniques [17], [18], [19], [20], [21]. Clinical decision making can be done based on 3D reconstruction created after segmentation of CT image data. Therefore, accuracy of segmentation is a determinant factor for the success of such cases. Selection of optimal procedure and armamentarium including screws and implants by the surgeon can be done using patient's 3D virtual construct. This preoperative planning can help to mitigate the risks associated with surgery [22].

Development in imaging and sophistication in 3D printing methods has helped in the evolution of preoperative surgical planning. Today anatomical models can be fabricated with much ease using 3D Printing/Additive manufacturing/rapid prototyping. [13,14,23,24]. In the present scenario, spinal surgeons rely heavily on pre-operative CT-scans, which provide an ideal opportunity to manufacture 3D printed models, resulting in improved outcomes, less repetitive scanning, hence decreased radiation exposure.

Application of 3D printing technology plays an important role in identification and measurement of ‘Cobb angle’ for complex vertebral deformation [23,25,26]. 3D Printed models can be a suitable alternative towards reduction of the risk associated with surgeries based on visual inspection [26]. According to current literature, there is no report or long-term randomised control studies to support the role of 3d printing in reducing the surgical risk for complex AIS cases. A repeatable method to fabricate highly accurate model, could enable widespread use in simulation and pre-operative planning minimising clinical risks. This could also enhance patient education, decrease operative time and prevent unanticipated problems. 3D printed models are expected to be the exact replica of targeted anatomy. However, achieving precision and accuracy with image processing along with suitable fabrication process also incur potential errors. Therefore, acceptable clinical tolerance in the fabricated models demand rigorous examination[27].

The present study is a case series evaluating image data from patients with varying degrees of scoliosis (Cobb angle varying from 40 to 92 degrees). The entire process from CT scan to 3D model is optimised. Further, the 3D printed spine models have been rescanned using CT and their accuracy has been tested. This study has the potential of improving presurgical planning for complex spinal surgery by accurate pedicle screw placement along with reduction in operative time and radiation exposure in spinal surgeries especially for young people. This challenge is still unaddressed in current literature.

## 2 Method and materials

A brief overview of the process from image acquisition to analysis has been documented in following sections.

### 2.1 3D model development

Anonymised CT-scan images (slice thickness: 1-2 mm; image voxel: 512×512×434) of five recruited patients, suffering from varying degrees of scoliosis were obtained from Royal National Orthopaedic Hospital, Stanmore, United Kingdom (ethical permission was obtained prior to the study; ethical permission reference no. 19/LO/1466). Informed consent was also obtained from the patients. The five CT-scan datasets used in this study consisted of two female and three male subjects (between 9-16 years age), with mean age of 13 years suffering from idiopathic scoliosis. These were chronologically ordered as, K1, K4, K7, K10 and K11, with Cobb angles of 39.78°, 95°, 49°, 51°, and 92° degrees, respectively (Table 1). There were multiple scoliotic curves in few of our cohort chosen for the present study, however, only one deformation curve was considered.

**Table 1:** Parameters of the patient CT data used in the study

Parameter	Spine ID (Anonymous patient identification)				
	K1	K4	K7	K10	K11
CT Scanner (Model, Software)	Phillips Ingenuity Core 128, v.4.1	Phillips Ingenuity Core 128, v.4.1	Phillips Ingenuity Core 128, v.4.1	Phillips Ingenuity Core 128, v.4.1	Siemens somatom Def AS, V.Syngo CT2012B
Series description	Axial ST iDose(4)	Axial Bone, iDose (3)	Axial Bone thin slices, idose(4)	Axial Media-Stinum, iDose (3)	Axial Biphasic AP2.0 Soft
Spinal region	Thoracic, Lumbar	Cervical, Thoracic, Lumbar	Thoracic Lumbar, Sacral	Thoracic	Thoracic
Age (yrs.)	14	16	13	13	9
Gender	Male	Male	Female	Female	Male
Image extent (Voxels)	512 x512x 498	512 x 512x 346	512 x 512x 348	512 x512 x281	512 x 512 x 376
In-Plane resolution (X×Y mm^2^)	0.4297 x 0.4297	0.6836x 0.6836	0.3906 x 0.3906	0.5918 x 0.5918	0.6074 x 0.6074
Z Spacing(mm)	0.5	1	1	1	1
Slice Thickness (mm)	1	2	2	2	2
No. of slices	498	364	348	281	376
Single collimation width (mm)	0.625	0.625	0.625	0.625	1.2
Acquisition type	Spiral	Spiral	Spiral	Spiral	Spiral

**Fig. 1:**
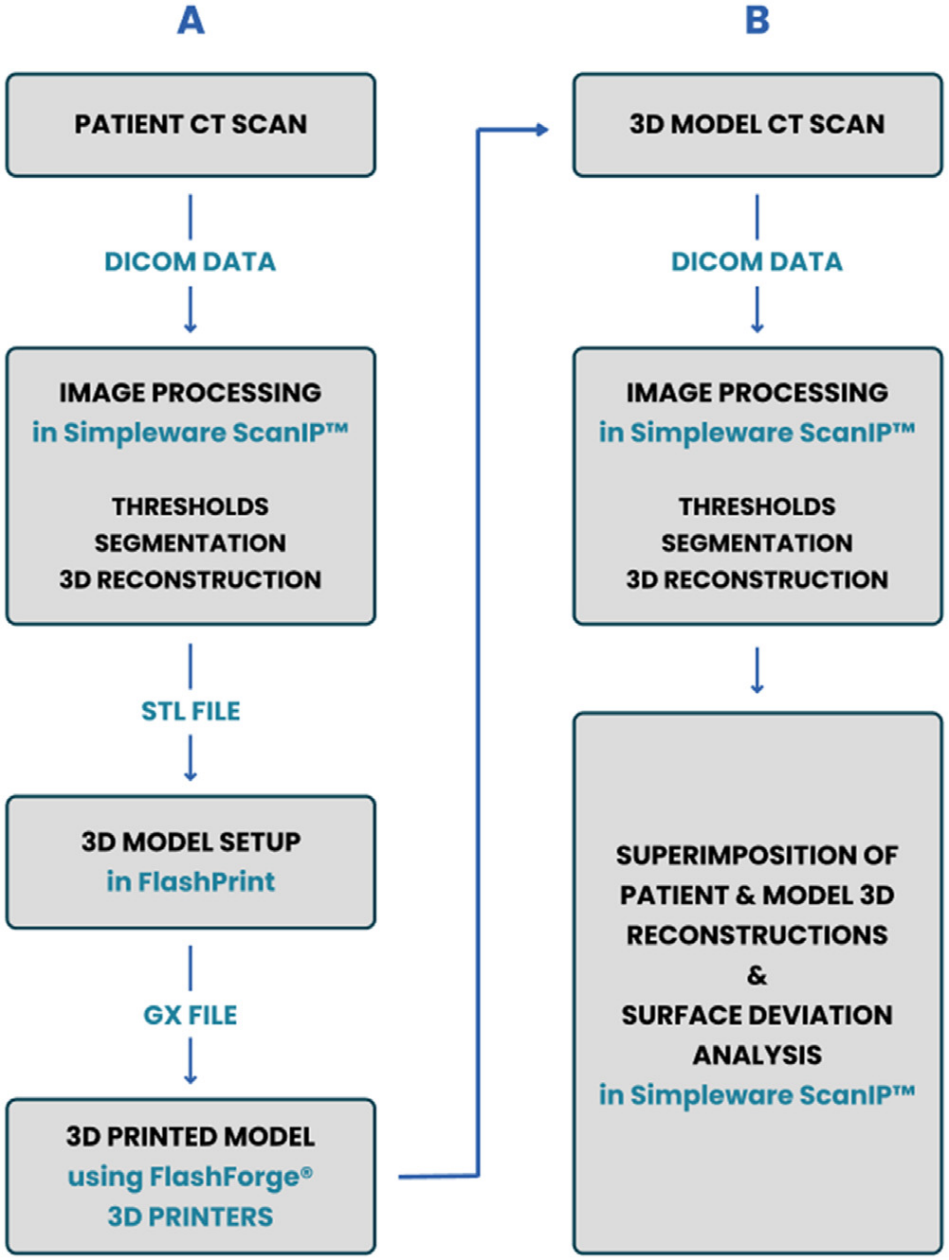
Schematic representation of the workflow: Column A represents the process used for 3D printing from medical CT imaging, and Column B demonstrates the additional steps to evaluate the accuracy of the 3D printed model

The 3D solid models of the patient's scoliotic spines were obtained using an image analysis software package from Simpleware ScanIP (Synopsys, Inc., UK). The process comprised of the following steps: segmentation of the CT-scan images based on Hounsfield Units (HU) of cortical bone (HU 100-2000), mask development [28], smoothing of the contours of each slice using manual segmentation, and preparing a solid model of the spine from the masks (Table 2). The segmentation and surface mesh quality were checked for irregularities, holes and overlapping edges. The segmentation of each spine data formed the ground-truth (models developed from the patients CT scan data) comparison for the corresponding 3D printed model segmentation, which was used to evaluate the accuracy of the 3D printing workflow. The methodology from data acquisition to generation of 3D printed model is described in Fig. 1. The created 3D surface model from patient CT-Scan was then exported for 3D printing in STL file format for 3D printing of the spinal models.

**Table 2:** Segmentation parameters for the patient CT and model CT, and 3D printing durations

Segmentation Parameters	Spine ID				
	K1	K4	K7	K10	K11
Patient CT					
Lower Threshold (HU)	176	109	230	170	125
Upper threshold (HU)	1415	1435	1308	1725	1933
Segmentation time	480 mins	510 min	210 min	120 min	1260
3D printing time	1740 mins	1860 mins	1200 mins	1500 mins	1260 mins
3D printed model CT					
Lower Threshold (HU)	-571	-571	-571	-571	-571
Upper threshold (HU)	970	970	970	970	970
Segmentation time	60 mins	40 mins	30mins	60 mins	60 mins
Total Processing Time	2280 mins	2410 mins	1440 mins	1680 mins	1485 mins

### 2.2 3D printing of the anatomical models

**Fig. 2:**
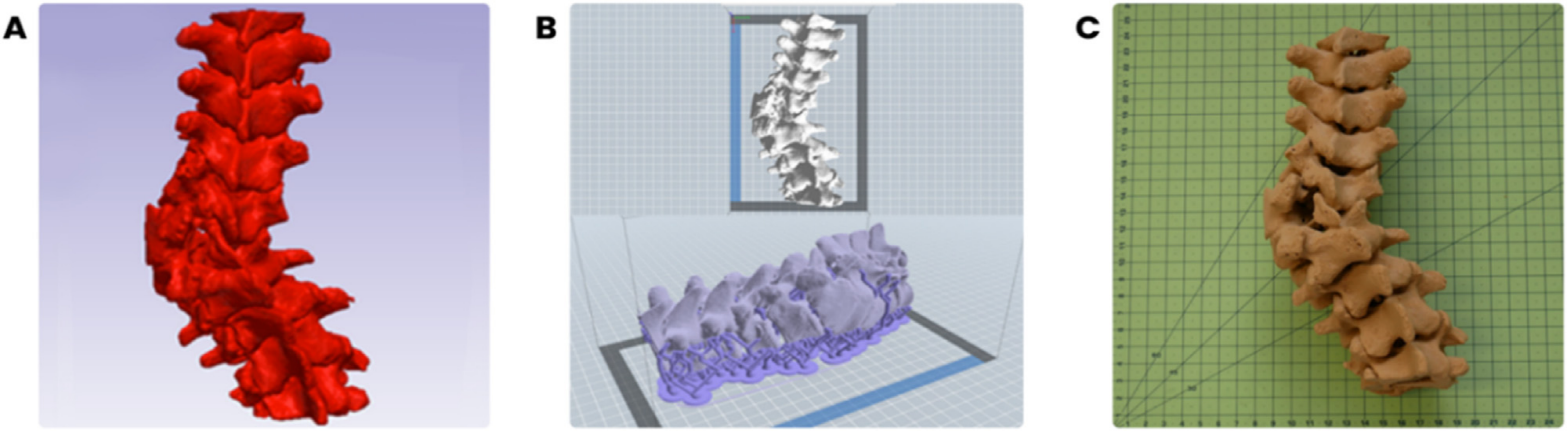
3D printed model workflow: (A) segmentation and 3D model reconstruction in Simpleware ScanIP, (B) 3D model setup and addition of raft and supports in FlashPrint, and (C) 3D printed model.

The STL file was pre-processed for 3D printing (Dreamer, Zhejiang Flashforge 3D technology Co., Ltd., China). Vertebral bodies of the models (Fig. 2) were printed using PolyWood (PolyMaker, China), where layer height was maintained at 0.18 mm, infill pattern was gyroid, print speed 50 mm/s, the extruder nozzle width was 0.4 mm, nozzle temperature was 220°C and the platform temperature was 55°C.

CT-scans were obtained for 3D Printed models. The same methodology (Section 2.1) was followed to generate virtual 3D masks of each printed model. Manual selection was required as the threshold ranges for segmentation were adjusted to accommodate for the difference in greyscale values (HU) as the 3D models were made of thermoplastics. The CT data was exported in DICOM file format and processed in Simpleware ScanIP (Synopsys Inc., UK). Once satisfied that the vertebral bone had been optimally isolated, a smoothing filter was applied only once using the recursive Gaussian tool with a 1-pixel radius to smooth the surface and minimise the effect on geometrical accuracy. The 3D printed models were visually inspected after printing for any obvious printing errors.

### 2.3 Surface deviation analysis of the 3D printed models

Simpleware ScanIP (Synopsis Inc., UK) software was used for superimposition and surface deviation analysis of the virtual segmentations of the patient and model. The original patient segmentation mask was isolated, and a model preview was generated for the highest quality. This was further converted from ‘Mask to Surface’ using the surface tools, and a new surface was generated. These steps were repeated for the segmentation mask of the 3D printed model.

**Fig. 3:**
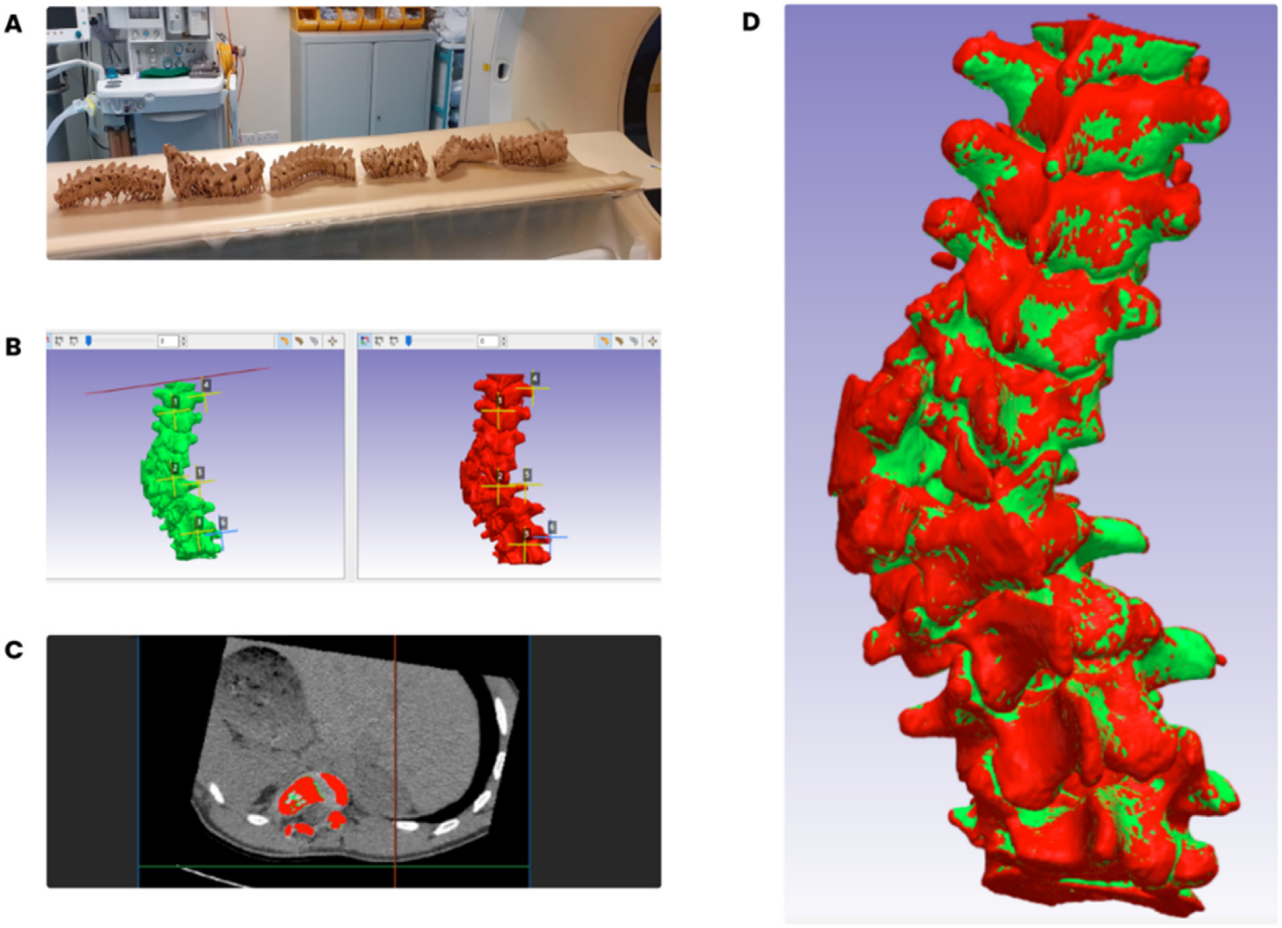
Superimposition and surface deviation analysis using Simpleware ScanIP: (A) CT-scan of 3D printed spinal models with region of deformity of interest, (B) six corresponding landmark placements on the patient ‘ground-truth’ (red) and model (green) virtual 3D reconstructions (C) axial view of superimposed images (D) final superimposition of 3D reconstructions

The two datasets (from the patient CT scans, and the scans from the 3D printed models of the scoliotics spines) were registered; the patient's surface was chosen as the ‘fixed reference dataset’ and the corresponding printed model surface for the corresponding registration dataset. For consistency, six corresponding landmarks were then manually selected on each surface, three in the sagittal plane and three in the coronal plane, in the upper, middle, and lower zones of the patient surface and corresponding model surface (Figure 3). This resulted in the root mean square error (RMSE) within the two consecutive anatomical models. The cobb angle of the scoliotic spines was measured by the surgical team and analysed further to identify any correlation to the RMSE errors from surface deviation analysis of the severely affected areas.

## 3 Result and discussion

This study evaluated accuracy of 3D printed spinal models from CT scan image of 5 patients with scoliosis and cobb angle varying between 40-95°. Accuracy of spinal model is paramount if it must be used by surgical team for pre/intra operative planning or surgical guide designs in complex scoliosis cases. As there was lack of evidence for accuracy of 3D printed spinal models, validation study was planned by performing a rescan of the 3D printed model using standard CT protocol and further reconfirming its accuracy, so that any variation during 3D printing process, use of materials etc. can be rigorously evaluated and optimised.

### 3.1 CT reconstruction of complex spine

Image acquisition and segmentation form the backbone in determining the accuracy of printed models. Image acquisition needs to be performed by software for medical purposes. This study used commercially available Simpleware ScanIP (Synopsys Inc., UK). Auto segmentation is helpful for most bone segmentation [[29], [30]], but complex structures demand manual segmentation. Knowledge of human anatomy helps to understand anatomical structures to perform complex segmentation such as in AIS [17]. Auto segmentation was checked manually for any inaccuracies and corrected, to ensure accuracy of segmentation process for complex spinal anatomies.

### 3.2 CT scanning of 3D printed models and their accuracy

Fused filament fabrication (FFF) printing method has been commonly cited for production of anatomical models using PolyWood plastic filament due to its relatively low cost and marked accuracy for medical modelling. [20,21,31]. FFF as a printing method provides print accuracy of 0.4 mm, thus can be accurately scanned using current CT protocol (using slices ranged from 0.5 to 1 mm)[32]. This does substantiate the use of (FFF) printing with PolyWood materials for fabrication of complex scoliosis models.

### 3.3 Accuracy of 3D printed model and correlation with cobb angle

**Fig. 4:**
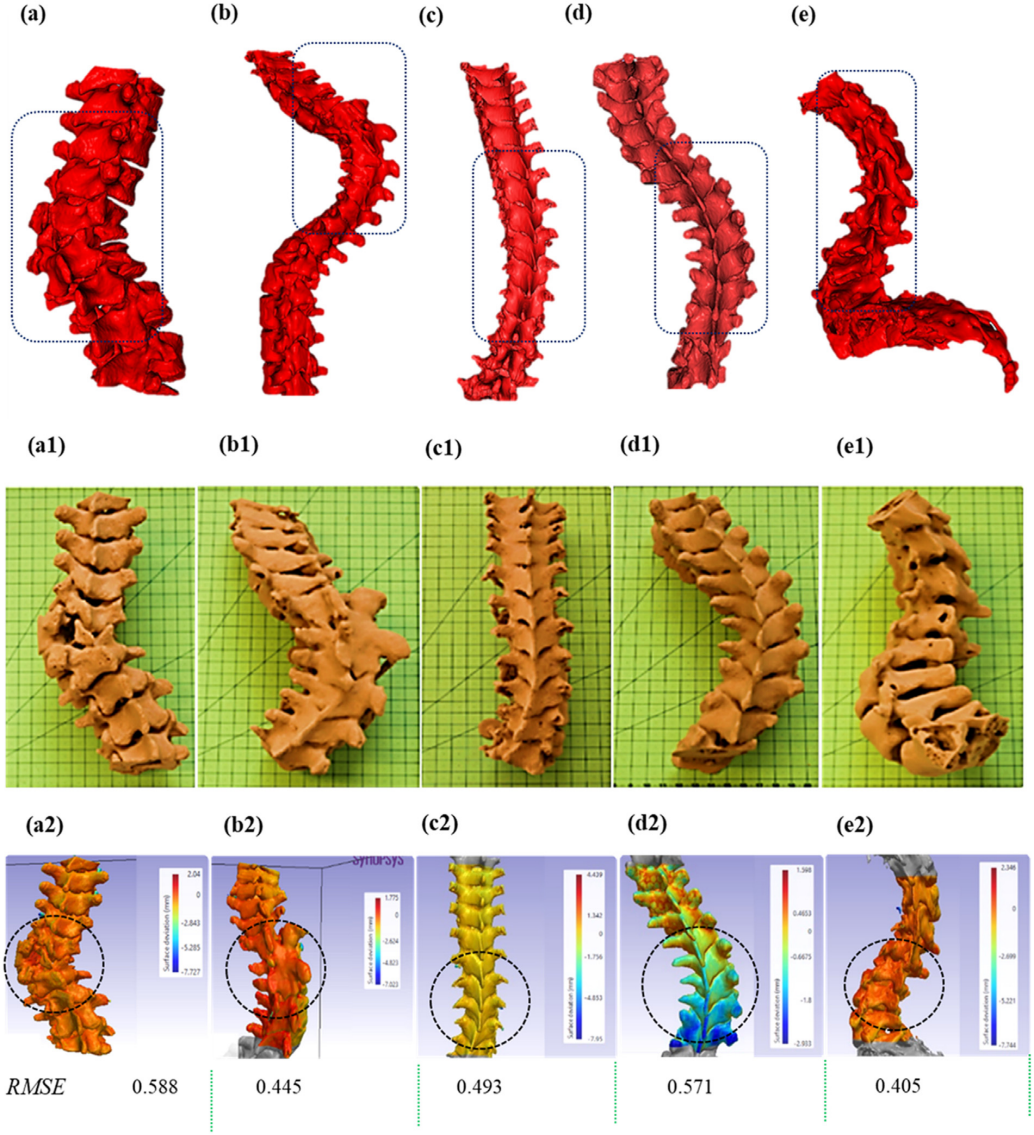
Analysis of accuracy of 3D reconstruction of the severe scoliotic spines: 3D reconstruction from corresponding CT scan dataset of patient (a) K1, (b) K4, (c) K7, (d) K10 and (e) K11; (a1 – e1)3D printed samples of the severely affected segments of the spines of the corresponding patients; (a2 – e2) surface deviation analysis of the 3D printed sections and the 3D reconstructed CAD files of the spines. *RMSE: Root Mean Square Error between the two consecutive surfaces (in mm); †Areas highlighted by dotted lines (a-e) showing the areas of severe deformations of the patients’ spines; †Areas encircled with dotted line(a2-e2) highlighting the severe areas.

Both the 3D models (Fig. 4 a-e), developed from ground-truth CT-scans of the scoliotic patients and the ones developed from re-scans of the 3D printed models (Fig. 4 a1-e1)were found to be accurate on visual inspection (Fig. 4). To establish a quantitative difference between ground-truth CT-scans and 3D printed spine models, reconstructed CT data from both were superimposed to calculate values of deviation and represented as the root mean square error (RMSE) values for individual patient spine. RMSE is a reliable measure for consideration of accuracy between 3D scans [18,[33], [34], [35], [36]]. These data were quantitatively represented as colour map as shown in (Fig. 4 a2-e2).

**Fig. 5:**
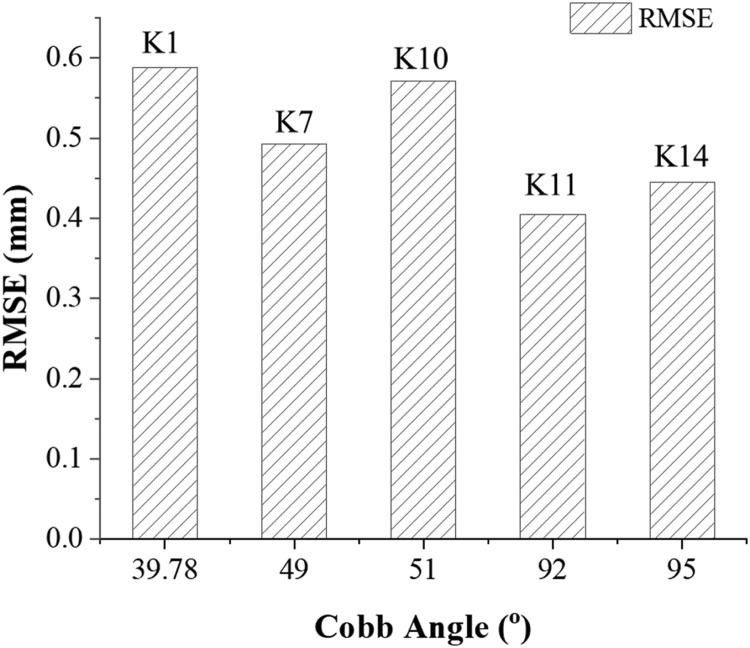
Influence of Cobb angle of the complex paediatric spines (*Spine ID: K1,K4,K7,K10,K11*) on the Root mean Square Error (RMSE) values of the surface deviation analysis between the 3D printed phantom and the spine CAD models

To establish correlation between complexity of spine and accuracy of corresponding 3D printed model, Cobb angle with increasing deformity for 5 patients was plotted against RMSE values as shown in Fig. 5. It is evident from Fig. 5 that despite the high inter-patient variability in terms of the complexity in the spines (represented by Cobb angle), the RMSE values are within the range of 0.4-0.6 mm. The overall mean RMSE was 0.5±0.07 mm and the average mean deviation for all five cases was very close to 0.1 mm. This indicates the reliability of the workflow in complex case scenarios. As suggested by Hicks et.al., 2010, the standard accepted encroachment level of the screws in a spinal surgery is in the range of ∼2 mm (REF: Hicks et al 2010), which support surgical accuracy established by our models.

The effect of scanning with different models of CT scanner using different parameters did not appear to affect or diminish the accuracy of the models produced. Moreover, the CT scan machines are supposed to be calibrated using phantoms to identify accurate HU values for the living tissues. The good agreement of the actual contours of the pathological spines, also served as an indirect measure of the optimal calibration, hence justifying the reliability of the machine.

In this study, the focus was scoliosis affecting the thoracolumbar spine. The present methodology could be implemented to evaluate other spinal pathologies. The unique, complex anatomy of the cervical vertebrae along with its interface with the skull is a challenging area to undertake surgical instrumentation. Prospective controlled studies with larger samples would help assess the validity and reliability of this method. Accurate and repeatable methods would help increase the potential uptake of 3D printing technology in the surgical community.

## 4 Conclusion

This work critically examined the capabilities of patient specific 3D printed spinal models for complex scoliosis surgery with Cobb angle varying from 40 to 95°. The work demonstrated that FFF based 3D printing workflow could be adapted for presurgical planning of complex adolescent scoliotic patients. This provides clinically acceptable level of accuracy for surgical planning and screw placements practice within 0.5 mm accuracy, shows promise for this technology adoption for safer surgical planning for complex AIS. The benefits and drawbacks for both patients and staff and the long-term clinical efficacy and safety of using 3D printed models need to be further evaluated if we are to see more widespread uptake. This would require larger patient cohorts and long-term studies to investigate this expanding clinical field.

### Research ethics

The study was conducted following ethical permission reference no. 19/LO/1466. Consent was also obtained for the use of the CT data in accordance with the study protocol.

## Declaration of Competing Interest

The authors declare that they have no known competing financial interests or personal relationships that could have appeared to influence the work reported in this paper.

## References

[1] Kuznia A.L., Hernandez A.K., Lee L.U. (2020). Adolescent idiopathic scoliosis: common questions and answers. Am Fam Physician.

[2] Tsirikos A.I., Roberts S.B., Bhatti E. (2020). Incidence of spinal deformity surgery in a national health service from 2005 to 2018: an analysis of 2,205 children and adolescents. Bone Jt Open.

[3] Perez-Machado G., Berenguer-Pascual E., Bovea-Marco M. (2020). From genetics to epigenetics to unravel the etiology of adolescent idiopathic scoliosis. Bone.

[4] Altaf F., Gibson A., Dannawi Z. (2013). Adolescent idiopathic scoliosis. BMJ.

[5] Garcia-Cano E., Arambula Cosio F., Duong L. (2018). Dynamic ensemble selection of learner-descriptor classifiers to assess curve types in adolescent idiopathic scoliosis. Med Biol Eng Comput.

[6] Campbell R.M., Smith M.D. (2007). Thoracic insufficiency syndrome and exotic scoliosis. J Bone Joint Surg Am.

[7] Anthony A., Zeller R., Evans C. (2021). Adolescent idiopathic scoliosis detection and referral trends: impact treatment options. Spine Deform.

[8] Jada A., Mackel C.E., Hwang S.W. (2017). Evaluation and management of adolescent idiopathic scoliosis: a review. Neurosurg Focus.

[9] Pan Y., Lu G.H., Kuang L. (2018). Accuracy of thoracic pedicle screw placement in adolescent patients with severe spinal deformities: a retrospective study comparing drill guide template with free-hand technique. Eur Spine J.

[10] Elliott M.J., Slakey J.B. (2014). CT provides precise size assessment of implanted titanium alloy pedicle screws. Clin Orthop Relat Res.

[11] Lu S., Xu Y.Q., Chen G.P. (2011). Efficacy and accuracy of a novel rapid prototyping drill template for cervical pedicle screw placement. Comput Aided Surg.

[12] Kepler C.K., Pavlov H., Kim H.J. (2012). Preoperative templating before spinal fusion using a fluoroscopic multiplanar imaging system is as accurate as CT scan and uses substantially less radiation. J Pediatr Orthop.

[13] Alpizar-Aguirre A., Cabrera-Aldana E., Rosales-Olivares L. (2018). A new technique of pedicle screw placement with the use of sequential multilevel navigation templates based on patient-specific 3D CT reconstruction model: applicability in spine deformity. Acta ortopédica mexicana.

[14] Lu S., Zhang Y.Z., Wang Z. (2012). Accuracy and efficacy of thoracic pedicle screws in scoliosis with patient-specific drill template. Med Biol Eng Comput.

[15] Ferrari V., Parchi P., Condino S. (2013). An optimal design for patient-specific templates for pedicle spine screws placement. Int J Med Robot.

[16] Sheehan D.D., Grayhack J. (2017). Pediatric scoliosis and kyphosis: an overview of diagnosis, management, and surgical treatment. Pediatr Ann.

[17] Courbot J.B., Rust E., Monfrini E. (2016). Vertebra segmentation based on two-step refinement. J Comput Surg.

[18] Cook D.J., Gladowski D.A., Acuff H.N. (2012). Variability of manual lumbar spine segmentation. Int J Spine Surg.

[19] Martin-Noguerol T., Paulano-Godino F., Riascos R.F. (2019). Hybrid computed tomography and magnetic resonance imaging 3D printed models for neurosurgery planning. Ann Transl Med.

[20] Ribera-Navarro A., Gibson A., Shenoy R. (2021). Critical analysis for a safe design of 3D printed Patient-Specific Surgical Guides (PSSG) for pedicle screw insertion in spinal deformities. Annals of 3D Printed Medicine.

[21] De Vega B., Navarro A.R., Gibson A. (2022). Accuracy of pedicle screw placement methods in pediatrics and adolescents spinal surgery: a systematic review and meta-analysis. Global Spine J.

[22] Archavlis E., Schwandt E., Kosterhon M. (2016). A modified microsurgical endoscopic-assisted transpedicular corpectomy of the thoracic spine based on virtual 3-dimensional planning. World Neurosurg.

[23] Wang Y.T., Yang X.J., Yan B. (2016). Clinical application of three-dimensional printing in the personalized treatment of complex spinal disorders. Chin J Traumatol.

[24] Yamaguchi J.T., Hsu W.K. (2019). Three-dimensional printing in minimally invasive spine surgery. Curr Rev Musculoskelet Med.

[25] Forsberg D., Lundstrom C., Andersson M. (2014). Model-based registration for assessment of spinal deformities in idiopathic scoliosis. Phys Med Biol.

[26] Mobbs R.J., Coughlan M., Thompson R. (2017). The utility of 3D printing for surgical planning and patient-specific implant design for complex spinal pathologies: case report. J Neurosurg Spine.

[27] Kanawati A., Fernandes R.J.R., Gee A. (2021). Geometric and volumetric relationship between human lumbar vertebra and ct-based models. Acad Radiol.

[28] Huang H., Xiang C., Zeng C. (2015). Patient-specific geometrical modeling of orthopedic structures with high efficiency and accuracy for finite element modeling and 3D printing. Australas Phys Eng Sci Med.

[29] Ruiz-Espana S., Domingo J., Diaz-Parra A. (2017). Automatic segmentation of the spine by means of a probabilistic atlas with a special focus on ribs suppression. Med Phys.

[30] Taddei F., Pancanti A., Viceconti M. (2004). An improved method for the automatic mapping of computed tomography numbers onto finite element models. Med Eng Phys.

[31] Ribera-Navarro A., Shenoy R., Cunningham G. (2021). Patient-specific 3D-printed surgical guides for pedicle screw insertion: comparison of different guide design approaches. J 3D Print Med.

[32] Searle B., Starkey D. (2020). An investigation into the effect of changing the computed tomography slice reconstruction interval on the spatial replication accuracy of three-dimensional printed anatomical models constructed by fused deposition modelling. J Med Radiat Sci.

[33] George E., Liacouras P., Rybicki F.J. (2017). Measuring and establishing the accuracy and reproducibility of 3d printed medical models. Radiographics.

[34] Decker S.J., Ford J.M. (2019). Forensic personal identification utilizing part-to-part comparison of CT-derived 3D lumbar models. Forensic Sci Int.

[35] Petropolis C., Kozan D., Sigurdson L. (2015). Accuracy of medical models made by consumer-grade fused deposition modelling printers. Plastic Surg.

[36] Mobbs R.J., Coughlan M., Thompson R. (2017). The utility of 3D printing for surgical planning and patient-specific implant design for complex spinal pathologies: case report. J Neurosurg-Spine.

